# Wound Healing Modulation through the Local Application of Powder Collagen-Derived Treatments in an Excisional Cutaneous Murine Model

**DOI:** 10.3390/biomedicines10050960

**Published:** 2022-04-21

**Authors:** Selma Benito-Martínez, Bárbara Pérez-Köhler, Marta Rodríguez, Jesús María Izco, José Ignacio Recalde, Gemma Pascual

**Affiliations:** 1 Departamento de Medicina y Especialidades Médicas, Facultad de Medicina y Ciencias de la Salud, Universidad de Alcalá, 28805 Alcalá de Henares, Spain; selma.benito@uah.es (S.B.-M.); barbara.perez@uah.es (B.P.-K.); 2 Biomedical Networking Research Centre of Bioengineering, Biomaterials and Nanomedicine (CIBER-BBN), 28029 Madrid, Spain; marta.rodriguezma@uah.es; 3 Ramón y Cajal Health Research Institute (IRYCIS), 28034 Madrid, Spain; 4 Departamento de Cirugía, Ciencias Médicas y Sociales, Facultad de Medicina y Ciencias de la Salud, Universidad de Alcalá, 28805 Alcalá de Henares, Spain; 5 Viscofan S.A., 31192 Tajonar, Spain; izcoj@viscofan.com (J.M.I.); recaldei@viscofan.com (J.I.R.)

**Keywords:** wound healing, collagen powder, tissue repair, regenerative medicine, excisional cutaneous model

## Abstract

Wound healing includes dynamic processes grouped into three overlapping phases: inflammatory, proliferative, and maturation/remodeling. Collagen is a critical component of a healing wound and, due to its properties, is of great interest in regenerative medicine. This preclinical study was designed to compare the effects of a new collagen-based hydrolysate powder on wound repair to a commercial non-hydrolysate product, in a murine model of cutaneous healing. Circular excisional defects were created on the dorsal skin of Wistar rats (*n* = 36). Three study groups were established according to the treatment administered. Animals were euthanized after 7 and 18 days. Morphometric and morphological studies were performed to evaluate the healing process. The new collagen treatment led to the smallest open wound area throughout most of the study. After seven days, wound morphometry, contraction, and epithelialization were similar in all groups. Treated animals showed reduced granulation tissue formation and fewer inflammatory cells, and induction of vasculature with respect to untreated animals. After 18 days, animals treated with the new collagen treatment showed accelerated wound closure, significantly increased epithelialization, and more organized repair tissue. Our findings suggest that the new collagen treatment, compared to the untreated control group, produces significantly faster wound closure and, at the same time, promotes a slight progression of the reparative process compared with the rest of the groups.

## 1. Introduction

Cutaneous wound healing is an important physiological process to restore the skin barrier after trauma. This process includes complex and dynamic pathways that are classified into three main consecutive but overlapping stages: the inflammatory, proliferative, and maturation/remodeling phases [1]. This involves the simultaneous actuation of soluble mediators, blood cells, the extracellular matrix, and epithelial and parenchymal cells [2].

The inflammatory phase occurs immediately after tissue damage; the hemorrhage triggers the coagulation cascade to restore hemostasis and prevent further blood loss. Hemostasis begins with the formation of a platelet plug, followed by a provisional fibrin matrix that will favor the migration of inflammatory cells. In addition to forming the clot, the thrombocytes secrete various mediators, growth factors and cytokines, which, together with the factors produced by the coagulation cascade itself, recruit inflammatory cells and activate tissue repair mechanisms [3,4].

Wounds that fail to progress satisfactorily through the normal phases of healing become chronic wounds, which represent a major problem to healthcare systems [5,6]. This may be due to a whole series of potential stimuli such as local tissue ischemia, necrotic tissue, bioburden, or even repeated trauma, which happens when wounds remain in the inflammatory phase, contributing to their chronicity [7]. However, this problem more frequently occurs in patients with underlying disorders such as peripheral artery disease, diabetes, venous insufficiency, and nutritional deficiencies, as well as other disease states [8,9].

These lesions show an altered and sustained inflammatory response, altered protease levels, and a deficient extracellular matrix, as well as impaired granulation tissue formation and maturation; in addition, the precise balance between the production and degradation of collagen is lost, and degradation dominates [10,11].

At both clinical and experimental levels, multiple therapies have been designed in attempts to improve healing. Among them, the use of collagen represents a good option for treating cutaneous wounds.

Collagen is a critical component of a healing wound, and the most abundant protein of the extracellular matrix of the dermal tissue. In addition to its important role as a scaffold in skin, this protein is of the utmost importance as a signaling molecule in the regulation of all stages of the reparative process. Regarding the inflammatory stage, it has been demonstrated that collagen breakdown products are chemotactic for a variety of cell types, including macrophages and fibroblasts, required for the formation of granulation tissue, enhancing phagocytosis and immune responses. During the proliferative phase, it promotes the growth of fibroblasts, which contribute to collagen deposition and granulation tissue formation, and keratinocytes in the wound. In the last phase, which is characterized by maturation and tissue remodeling, immature type III collagen is replaced by mature type I collagen, which provides tensile strength to the newly formed tissue [7].

Collagen is of great interest in regenerative medicine due to its low immunogenicity, remarkable biocompatibility, ease of application, and good biodegradability. Additionally, collagen-based treatments have the ability to absorb wound exudates and maintain a moist environment, which is essential for an optimal healing process. As a biodegradable component, they do not require removal from the wound bed before re-application, preventing loss of fluid and protecting the wound from bacterial infection and other agents [12]. These properties make collagen an ideal wound therapy agent to improve and accelerate the healing process. Currently, the main sources of collagen are typically bovine, equine, avian, or porcine, although alternative natural (marine) or engineered (recombinant from plant or bacterial material) sources have been considered [13]. Numerous studies have assessed the collagen formats in wound healing; some of them use collagen as scaffolds/matrices [14], sponges [15], hydrogels [16], or powders [17].

Collagen-based wound products can be classified into two categories: native collagens, decellularized but maintaining ECM structure, and denatured collagens that are highly modified and have lost their three-dimensional structure [7,18]. Collagen powder is well known for promoting cellular recruitment and inflammation phase activation and exerting its activity immediately upon application, as compared to 3D scaffolds. A pilot study demonstrated that treatment of a full-thickness wound with collagen powder enhanced the maturity and strength of wound healing [19].

A variety of commercial collagen powder products are available on the market, varying in source, purity, manufacturing, and particle size. In some cases, native collagen can be hydrolyzed to produce low-molecular-weight peptides. Denaturation of native collagen produces three α chains in their random coiled form. Once the chains are separated, hydrolysis is carried out by the action of proteolytic enzymes (alcalase, papain, pepsin, and others). The resulting product is commonly called hydrolyzed collagen. It is composed of small peptides with low molecular weight 3–6 KDa [20]. The advantages of hydrolyzed collagen are that it avoids breakdown by endogenous enzymes, and is highly soluble and easily absorbed by the human body, allowing for immediate signaling. A study carried out in dogs [21] demonstrated that hydrolyzed collagen powder enhanced the percentage of epithelialization after seven days of treatment compared to the control.

Other commercial collagen-based products, derived from bovine cartilage in the form of powder, have been shown to be effective in the treatment of wounds by secondary intent such as pressure ulcers, venous stasis ulcers, and diabetic ulcers, as well as second-degree burns, post-radiation dermatitis, and wounds unresponsive to conventional treatments [22].

Researchers and companies have developed and marketed a wide variety of products presenting different compositions, but all focused on promoting wound healing.

Taking into account all these factors, the present preclinical study was designed to compare the effects on wound repair of two collagen-based powder products, a new hydrolyzed bovine dermal collagen powder (not yet on the market) and a non-hydrolyzed commercial collagen derived from bovine cartilage, in a murine model of cutaneous healing. The reparative process was assessed with respect to the evolution over time of the defect and inflammatory response, and the formation and maturation of new tissue.

## 2. Materials and Methods

### 2.1. Experimental Animals and Ethics

Female Wistar rats (*n* = 36) weighing around 250 g were used. The care of the animals used in this study and the experimental procedures were in accordance with current protocols on the use of animals in experimentation (European Directive 2010/63/EU, European Convention of the Council of Europe ETS123 and Spanish Royal Decree 53/2013), and the study was approved by the Animal Experimentation Ethics Committee of Universidad de Alcalá, Spain. In this study, a rat model of wound healing was developed to evaluate the effect of different collagen treatments on the tissue reparative process.

Animals were individually housed under controlled temperature and illumination conditions, with a complete diet (Harlan Laboratories, Houston, TX, USA) and water ad libitum.

### 2.2. Study Groups

The animals were randomly distributed into three study groups (*n* = 12), according to the treatment administrated:

– Control (*n* = 12): Rats without treatment

Powder treatment:

– T1 (*n* = 12): rats treated with Catrix^®^ (Lescarden Inc., New York, USA).

Catrix^®^ is a bovine cartilage collagen powder. The particles are composed of natural macromolecules (particle size 35 μm) of collagen arranged in the form of a three-dimensional network.

– T2 (*n* = 12): rats treated with collagen developed by Viscofan, S.A (Tajonar, Navarra, Spain).

This is a hydrolyzed bovine dermal collagen powder, mainly containing low-molecular-weight (3 KDa) type I peptides. These collagen peptides contain a unique composition and a high number of essential amino acids, such as proline, hydroxyproline, and glycine.

### 2.3. Surgical Technique and Sample Collection

Excisional wounds on the dorsal surface in rats is one of the most commonly used and standardized wound healing models [23,24]. These wounds are generated by the surgical removal of all skin layers (epidermis, dermis, and subcutaneous tissue) from the animal. This model allows the investigation of the inflammation, granulation tissue formation, reepithelialization, angiogenesis, and remodeling process.

**Figure 1:**
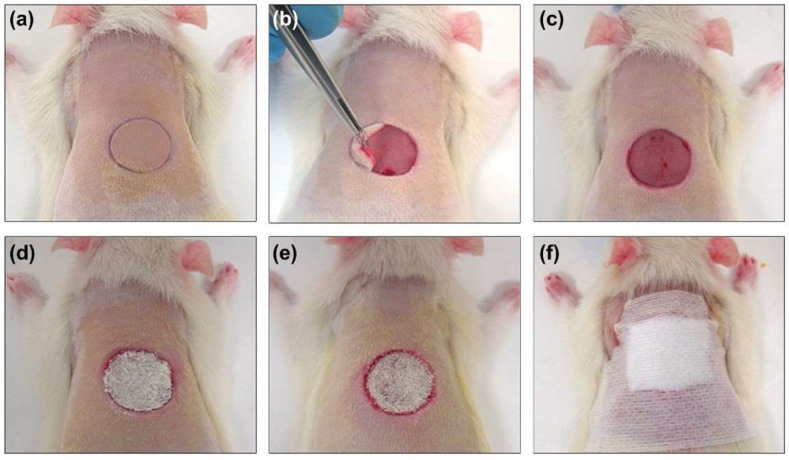
Surgical procedures and treatment application. (**a**,**b**) Excisional defect (1.5 cm diameter) created in the dorsa of the animals. Treatment was applied in the different study groups: (**c**) control group, (**d**) T1 group, and (**e**) T2 group. (**f**) The wound areas of all animals were covered with sterile dressings.

The animals were anesthetized in an inhalation chamber with a mixture of isoflurane (Forane^®^; AbbVieS.L.U., Madrid, Spain) at 4–5% and oxygen at a flow rate of 0.6–0.7 L/min. During the surgery, anesthesia was maintained by a face mask connected to a calibrated vaporizer, providing an inhalation dose of isoflurane of 2.5–3%. The fur on the backs of the rats was shaved with an electric razor, and the skin was disinfected with iodopovidone. After anesthesia, a circular (1.5 cm diameter) full-thickness defect, previously marked by a calibrated metallic punch and centered approximately 0.5 cm caudal to the scapulae, was created (Figure 1). The skin was removed with a scalpel and surgical scissors, following the previously marked line. The animals did not require postoperative analgesia. To minimize stress caused by the surgical procedure and individual housing, the animals were in visual, auditory, and olfactory contact with other rats, and environmental enrichment was provided every week.

Once the wound was created, the corresponding treatment was administered to all groups except the control group. Treatments were applied at 0, 3, 5, 7 and 9 days, covering the area with wound-protective devices (Figure 1). The scab covering the wound was removed before each treatment application to ensure its accessibility in the open area, and no debridement of the wound was performed in any case. The same procedure was carried out in the control group.

At the end of the established study times, the animals were euthanized in a CO_2_ inhalation chamber. The scar tissue was photographed for morphometric evaluation and subsequently excised. The tissue samples were sectioned into two halves transverse to the body axis for histological processing.

### 2.4. Morphometric Studies of Wound Evolution

Following surgery, animals were regularly weighed and monitored daily to evaluate the evolution of skin scarring. Immediately following surgery, during the study and at the end of the established study times, measurements of the defect were performed.

**Figure 2:**
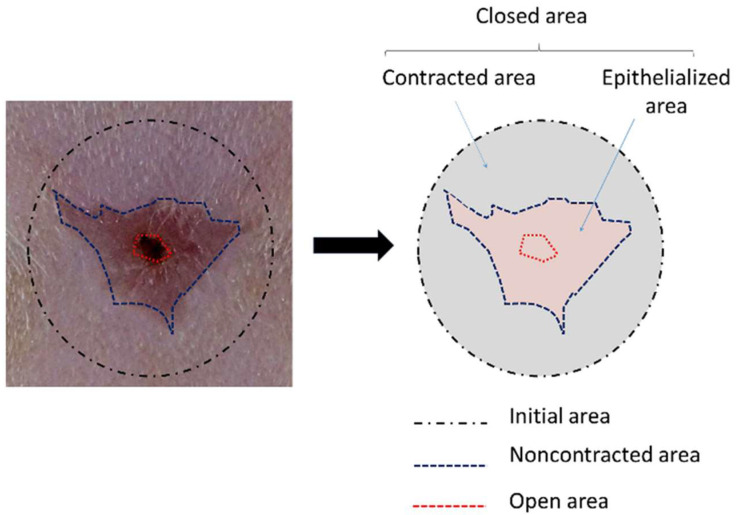
Morphometrical analysis of the wound. Photographs were taken at different study times and at the time of euthanasia. Measurements of initial, non-contracted, and non-closed areas of each animal, using an image analysis program, were used to calculate the areas covered by the epithelialization process (non-contracted area—open area) and contraction process (initial area—non-contracted area) as well as the relative contribution of these process to the wound closure, expressed as a percentage over the initial area of the defect.

To evaluate the wound closure after the defect was performed and at the time of euthanasia, cenital photographs, from a plane just above the experimental animals, of the defects and scar tissue were taken with a ruler for calibration. For this assessment, the initial area of the defect caused at the time of surgery (diameter: 1.5 cm), the area of the defect that remained unclosed after 7 and 18 days, and the contraction area were measured using ImageJ software (National Institutes of Health, Bethesda, MD, USA) (https://imagej.nih.gov/ij). These measurements were used to calculate the relative values of the processes of wound closure, epithelialization, and contraction (Figure 2).

### 2.5. Morphological Studies

For light microscopy analyses, the tissue samples obtained were fixed with solution F13 (60% ethanol, 20% methanol, 7% polyethylene glycol, and 13% distilled water) and paraffin-embedded. Tissue blocks were cut with a Microm HM-325 microtome (Microm International GmbH, Walldorf, Germany) into 5-μm-thick sections and placed onto slides coated with 0.01% polylysine (Sigma-Aldrich, St. Louis, MO, USA). Finally, the sections were dewaxed, rehydrated, and stained with hematoxylin–eosin and Masson’s trichrome (Goldner–Gabe variant), and Sirius red. Samples were examined under a Zeiss Axiophot light microscope (Carl Zeiss, Oberkochen, Germany). For each staining, six sections from the central zone of wound and three sections of the marginal edge were analyzed.

Both hematoxylin–eosin and Masson’s trichrome staining allowed for the general observation of the repairing tissue, granulation tissue, distribution of collagen inflammatory cells, and neoformed vessels. Sirius red staining was utilized to evaluate the organization and maturation of collagen fibers in the repairing tissue. Despite the lack of complete specificity, type I collagen (mature) appears as a reddish-orange stain, while type III collagen (immature) takes on a yellowish-green stain when observed under the polarized light microscope [25]. Morphological examination was performed by two independent histologists in a blinded fashion.

### 2.6. Statistical Analysis

The data were expressed as the mean ± standard deviation. To compare different study groups, the Mann–Whitney U test was used. All statistical tests were performed using the software package GraphPad Prism 5 (GraphPad Software Inc., San Diego, CA, USA). Significance was set at *p* < 0.05.

## 3. Results

### 3.1. Postoperative Follow-Up

All animals completed the assigned study time; only one from the control group was excluded due to evidence of self-harm near the area of the defect. This animal was excluded from subsequent macro and microscopic assessments.

On day 7, animals from all the groups exhibited the expected initial weight loss, although this was not significant with respect to the control group. On day 18 post-surgery, most of the groups displayed a weight gain.

### 3.2. Morphometric Results

#### 3.2.1. Wound Closure Evolution

**Figure 3:**
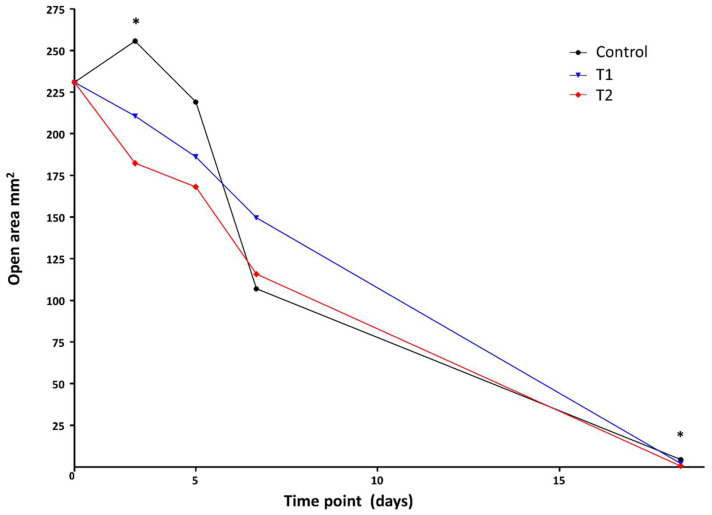
Evolution of the unclosed area of the defect over time. On day 3 post-surgery, statistical differences were observed between the control and T2 treatment (* *p* < 0.05). At days 5 and 7, no significant differences were detected between groups. At day 18, statistical differences were observed between the control and T2 groups (* *p* < 0.05).

For this assessment, the initial mean wound surface area at the time of surgery (diameter: 1.5 cm) was slightly increased (230.9 ± 28.01 mm^2^) compared to the theoretical area (176.7 mm^2^) (Figure 3).

On day 3 post-surgery, animals from the control group showed a higher wound distension of area defects than the T1 and T2 groups, which showed a reduction in the open area of the wound, this being more important in T2. The control group showed a statistically significant difference (255.71 ± 69.33 mm^2^) compared to the T2 group (182.30 ± 25.29 mm^2^); however, no differences were shown versus the T1 group (210.60 ± 62.81 mm^2^).

Five days after surgery, the wound distension was maintained (219.09 ± 59.04 mm^2^) in the control group, and no significant differences were detected between the groups.

On day 7, the open area had decreased and showed similar values in all groups, with no significant differences between them.

On day 18 post-surgery, all open areas from the different groups showed considerably reduced values, in all cases less than 10 mm^2^. At this time point, significant differences were also found between the control and T2 groups (5.38 ± 4.37 and 0.55 ± 0.66 mm^2^, respectively) (Figure 3).

#### 3.2.2. Wound Contraction and Epithelialization

**Figure 4:**
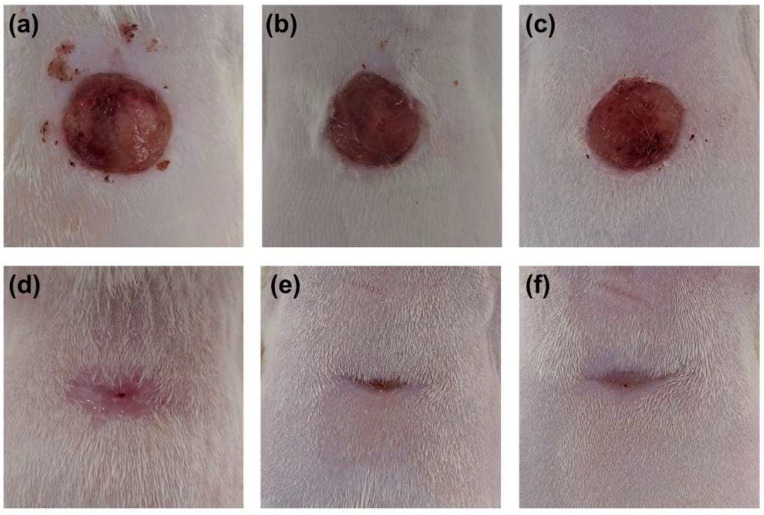
Macroscopic images show the appearance of the defects on (**a**–**c**) day 7 and (**d**–**f**) day 18 after treatment at the time of euthanasia. (**a**,**d**) Control group, (**b**,**e**) T1 group, and (**c**,**f**) T2 group.

**Figure 5:**
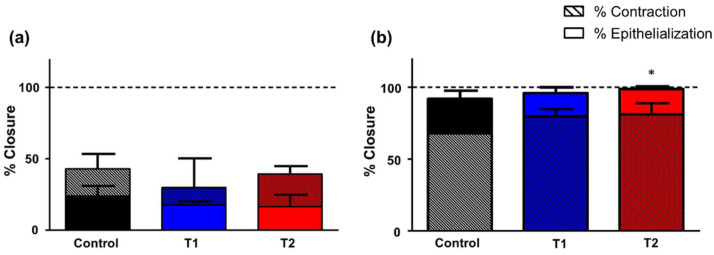
Morphometrical analysis of the defect on (**a**) day 7 and (**b**) day 18. Graphical representation of the percentage of wound closure expressed as mean % of the initial wound area covered ± SD by the contraction and epithelialization processes. On day 7 (**a**), no significant differences were observed in the percentage of epithelialization and contraction between any of the study groups evaluated. Regarding the contraction process, on day 18 (**b**), no significant differences were observed between groups; in the case of the epithelialization process, statistical differences emerged between the control and T2 group (* *p* < 0.05).

Seven days after surgery, the open surface had decreased with respect to the initial area, although this area was still considerable (Figure 4). The analysis of the evolution of the wound closure process revealed a greater proportional contribution of contraction over epithelialization (Figure 5a).

The highest contraction values were observed in the control group (42.91% vs. T1: 29.96% and T2: 39.30%) although no significant differences were observed.

Regarding the epithelialization process, no significant difference was observed between groups; mean values did not reach 25% of the initial area in all cases (Figure 5a). However, it must be taken into account that these values are in percentages and, in the groups in which less contraction occurred, there would be more surface to epithelialize.

Eighteen days after surgery and treatment, the open area was diminished with respect to after seven days. Macroscopically, in the majority of the groups, a centripetal pattern of closure was observed; the wound tended to close preferentially in the transversal axis (Figure 4).

The analysis of the evolution of the wound closure process revealed, contrary to after seven days, higher epithelialization than contraction values. The contribution of shrinkage was very important in all study groups, with mean values above 65%, reaching almost 85% of the initial area, although no significant differences were detected between the various groups.

Regarding the percentage of epithelialization, animals without treatment showed significantly lower values (91.64%) than animals with T2 treatments (98.47%) (*p* < 0.05) (Figure 5b).

### 3.3. Morphological Results

#### Evaluation of the Reparative Process

**Figure 6:**
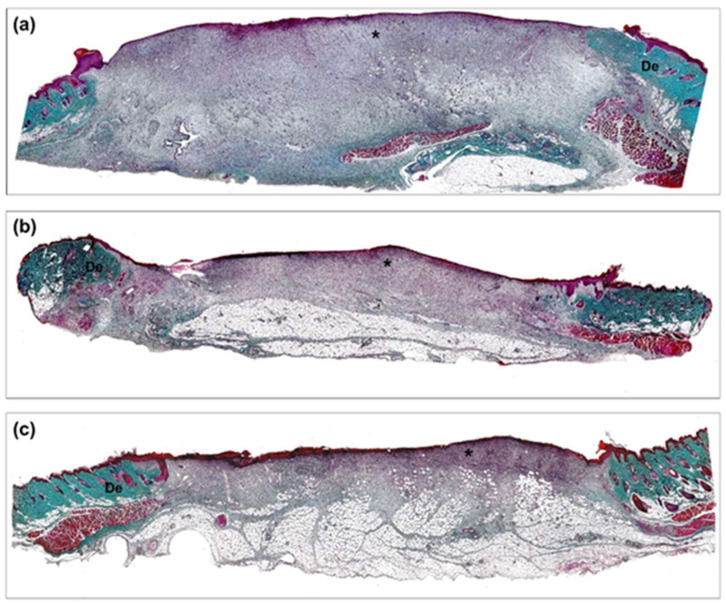
Histological evaluation. Panoramic view of the wound area seven days after treatment application (Masson’s trichrome, ×50). (**a**) Control group, (**b**) T1 group, and (**c**) T2 group. De: dermis; *: Neoformed granulation tissue.

Seven days after the intervention, all the animals showed a large nonepithelialized defect area and an inflammatory exudate covering the superficial zones. In general, in the wound area, granulation tissue with numerous blood vessels and signs of evident inflammatory and proliferative phases of reparative process were observed in panoramic histological images (Figure 6). The control group showed the greatest development and thickness of this tissue (Figure 6a). In groups T1 and T2, reduced granulation tissue formation with homogeneous thickness, fewer inflammatory cells, and induction of vasculature in the neoformed tissue were observed (Figure 6b,c).

**Figure 7:**
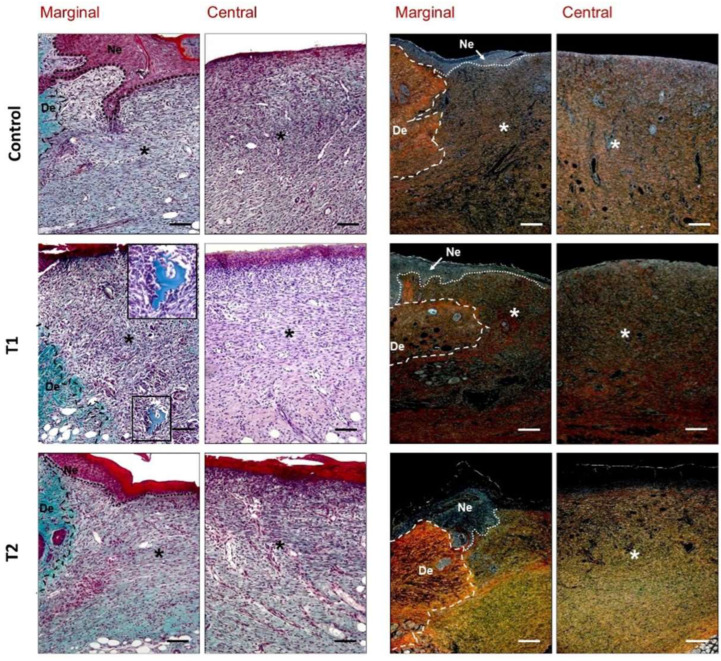
Histological evaluation of the different study groups. Details of the marginal and central zones of the wound seven days after treatment (Masson’s trichrome, hematoxylin–eosin, two columns on the left, and Sirius red ×160, two columns on the right). Type III collagen (immature) appears as a yellowish-green shade, while type I collagen (mature) takes on a reddish-orange stain. The boxed and magnified area (×320) showed that inflammatory cells associated with T1 treatment remain embedded in the repair tissue. De: dermis; *: wound zone; Ne: neoepidermis. Scale bar: 200 μm.

At a higher magnification, tissue edges adjacent to the wound showed an active and thickened epithelium with signs of cell proliferation and migration, covering neoformed granulation tissue and advancing between this and inflammatory exudate, compared to nondamaged epithelium (Figure 7). In these sections, it was possible to observe the border between the undamaged area showing dense dermal tissue and hair follicles, and the damage area with high inflammation and low collagen deposition (Figure 7).

Animals from the control group showed accumulation of inflammatory cells and myofibroblasts in a loose and poorly organized extracellular matrix with evident immature collagen deposition, revealed by the yellow color of the Sirius Red stain (Figure 7). In animals from the T1 group, moderate signs of inflammation were observed in the superficial layer of neoformed tissue. In five animals, some remains of the treatment immersed in the repair tissue were observed, around which inflammatory-type cells were also located (Figure 7). Repair tissue appeared more organized and the synthesis of new matrix with collagen type I deposition was observed in deeper areas. The superficial layer of granulation tissue in the T2 group showed a slightly increased number of inflammatory cells. Beneath this layer, the repair tissue exhibited good organization and a large deposit of immature collagen (type III) (Figure 7).

**Figure 8:**
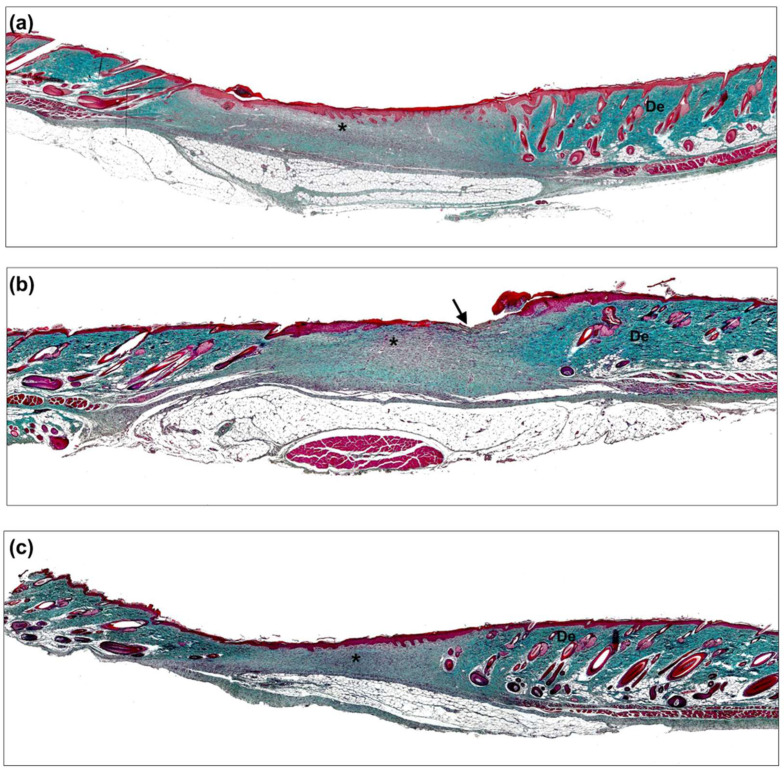
Histological evaluation. Panoramic view of the wound area 18 days after treatment application (Masson’s trichrome, ×50). (**a**) Control group, (**b**) T1 group, and (**c**) T2 group. De: dermis; *: Neoformed tissue; →: nonepithelized area.

Eighteen days after surgery, samples exhibited a significant approximation of the wound edges due to tissue contraction, as well as features of the initial stages of maturation and remodeling. Almost all the animals showed completely epithelialized wound areas; however, the control group displayed a higher mean open surface wound area (Figure 8). Panoramic histological images also showed some open area in the T1 group (Figure 8b).

**Figure 9:**
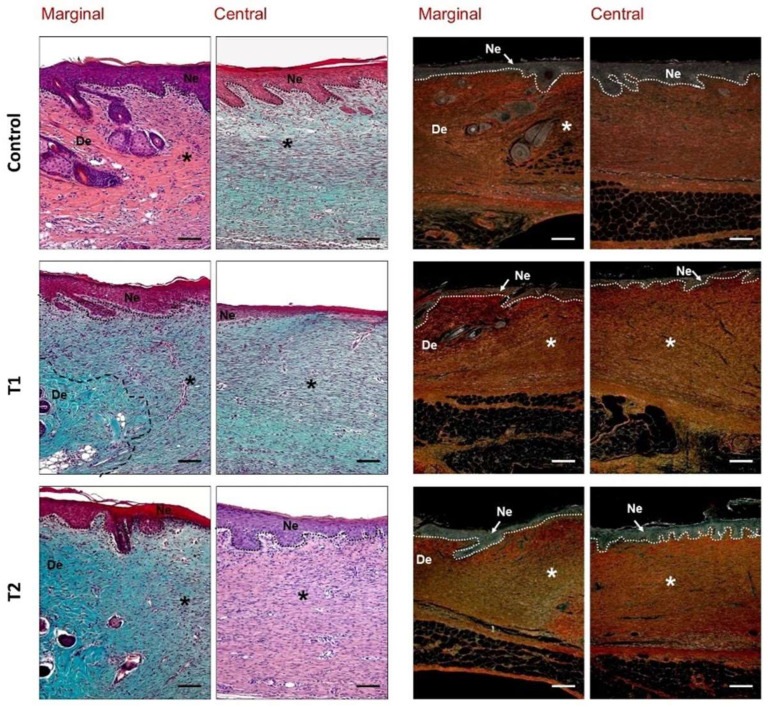
Histological evaluation of the different study groups. Details of the marginal and central zones of the wound 18 days after (Masson’s trichrome, hematoxylin–eosin, two columns on the left, and Sirius red ×160, two columns on the right). Type III collagen (immature) appears as a yellowish-green shade, while type I collagen (mature) takes on a reddish-orange stain. De: dermis; *: wound zone; Ne: neoepidermis. Scale bar: 200 μm.

In general, all study groups showed an advanced stage of inflammatory remission as well as progressive tissue maturation with reduced cellularity versus after seven days due to vascular retraction, decreased inflammation, and a low number of fibroblasts. Repaired tissue appeared more organized than after seven days, showing a significantly higher density of collagen with a more homogeneous distribution in the neoformed tissue. In some zones, fibroblasts and a fibrillar matrix were observed to be laid down parallel to the wound area, sealing the wound edges. In the tissue edges, adjacent to the uninjured area, enhanced collagenization of the neoformed connective tissue was observed (Figure 8 and Figure 9).

The control group presented a thicker neoepithelium than unwounded zones, where nontypical epidermal ridges invading the dermis were observed (Figure 9). The presence of inflammatory zones could be observed in the central portion of the wound that had not yet healed, where accumulations of inflammatory cells were present in the superficial and basal bands of the neodermis (Figure 8a and Figure 9).

T1 was the group where epithelial development was the most delayed. Some nonepithelialized zones in the central region of the defect or with the absence of any epidermal layer could be observed; however, the dermis exhibited good collagenization and some inflammatory cells that looked similar to those of previous groups (Figure 8b and Figure 9).

The greatest wound closure and epithelialization areas were observed in the T2 group. The epidermis was completely differentiated and stratified, including stratum corneum and active epithelial desquamation (Figure 9). The development of typical epidermal ridges alternating with well-collagenized dermal papillae could be observed. The dermis was dense and rich in collagen type I, and exhibited organized connective tissue with abundant fibroblasts and scarce inflammatory components (Figure 8c and Figure 9).

## 4. Discussion

The main objective of the wide range of devices or treatments developed to promote wound healing is that this physiological process promotes wound closure as soon as possible and results in a functionally and aesthetically satisfactory scar. For this purpose, the organism must be able to reduce cutaneous discontinuity by generating new granulation tissue in the wounded area. An epithelial barrier must be able to develop on this tissue, reaching continuity and avoiding re-openings through the formation of a resistant extracellular matrix. This results from cell proliferation, synthesis, and maturation of the extracellular matrix [3].

In general, the wound repair process occurs in almost all tissues after exposure to a destructive stimulus. It must be taken into consideration that, in this study, the physiological healing process was not compromised in any way. All the animals used were healthy animals without pathologies that might have affected healing; therefore, the model showed a standard healing pattern, with characteristics of the three phases of the skin repair process (inflammation, proliferation, and remodeling/maturation) appearing in a progressive and orderly manner until the achievement of newly formed connective tissue with reduced cellularity and a dense and organized extracellular matrix. With these requirements in mind, the treatments’ ability to improve wound closure were evaluated.

Although the study and knowledge of wound healing in humans have advanced considerably, the difficulties and limitations make the use of experimental models necessary for research in this field. For decades, the development of multiple animal models has been used, including dorsal wounds in small rodents, rabbit ear defects or porcine models [26,27]. However, the most used models for the study of skin repair are based on rats and mice due to their easy handling and maintenance. It must be taken into account that the use of these animals has some limitations due to differences in healing mechanisms compared to humans. One of them is the greater proportional contribution of the contraction process over epithelialization that occurs in rodents [27]. Excisional wounds are one of the most commonly used wound healing models because they allow the investigation of inflammation, granulation tissue formation, re-epithelialization, angiogenesis and the remodeling process [28].

Based on our group’s previous experience [29], we developed a murine model to evaluate both the initial phases of healing (7 days) and tissue remodeling (18 days) by using an excisional model. In the present experimental study, evidence for collagen powder as an adjunctive therapy in wound healing has been provided. We used one collagen-based product available on the market and obtained from the bovine tracheal cartilage. Catrix^®^ was approved by the FDA in 1998, and is frequently used in wound care. Several studies have indicated that this collagen powder could be an optimal agent to stimulate the wound healing process [22]. Clinical evidence from a prospective multicenter study treating resistant pressure ulcers showed a statistically significant improvement in complete healing using this product compared to untreated controls [30].

For these reasons, in this study we chose Catrix^®^ as a reference product to assess the performance of a new collagen-based product in a rat wound healing model. 

In the classic stages of wound repair, inflammatory phase occurs immediately after tissue damage with the development of a platelet plug and a provisional fibrin scaffold that promotes migration of inflammatory cells [3,4]. New tissue formation occurs 2–10 days after injury and is characterized by cellular proliferation and the migration of different cell types [3]. The first event is the migration of keratinocytes to the injured dermis, as we observed in our study groups after seven days. Tissue edges adjacent to the wound showed an active and thickened epithelium, with signs of cell proliferation and migration; neoformed granulation tissue showed signs of inflammatory and proliferative phases of the reparative process and important angiogenesis. The most important positive regulators of angiogenesis are vascular endothelial growth factor A (VEGFA) and fibroblast growth factor 2 (FGF2; also known as bFGF) [4].

The results of our study revealed, after seven days, some differences between the groups. In our T1 and T2 groups, reduced granulation tissue formation with homogeneous thickness, fewer inflammatory cells, and the induction of vasculature in the neoformed tissue were observed with respect to untreated animals, which exhibited more inflammation and less organized repair tissue. Some other findings indicate that the application of pure undiluted bio-collagen extracted from bovine skin dramatically improved wound healing in rats after seven days in terms of collagen production, wound filling, and the migration and differentiation of keratinocytes, being three times more effective than the commercial Catrix^®^ [31].

In the later part of this stage, fibroblasts, which are attracted from the edge of the wound, are stimulated by macrophages, and some differentiate into myofibroblasts [32], contractile cells that, over time, bring the edges of a wound together and are responsible for the process of wound contraction. Some authors, trying to minimize the important process of wound contraction that occurs in rodents to replicate human physiology, have described models of wound healing utilizing wound splinting, verifying a greater deposition of granulation tissue and not being affected by the rate of re-epithelialization [33]. In our study, the evolution of the wound closure process revealed a greater proportional contribution of contraction over epithelialization. The highest contraction values in our study were observed in the control group compared to the rest of the groups, although no significant differences were observed between them.

Fibroblasts and myofibroblasts interact and produce an extracellular matrix, mainly in the form of collagen, which ultimately forms the bulk of the mature scar [34]. Evident immature collagen type III deposition was observed in the repair tissue in our model as the main component of the fibrillar matrix.

The third stage of wound repair—remodeling—begins 2–3 weeks after an injury and lasts for a year or more. Eighteen days after surgery, samples exhibited a significant approximation of the wound edges due to tissue contraction. At the end of the study, all groups showed similar macroscopic closures, except animals treated with the new collagen (T2 group), which showed accelerated wound closure compared to the untreated group. Despite making a limited contribution to total closure in the present model, epithelialization showed an improvement in this group of animals.

The effects of bovine collagen-derived powder treatments, similar to those applied in our T2 group, were evaluated on the healing of open wounds in healthy dogs [21] and, according to our results, improved wound epithelialization.

During this last stage of healing, all the processes that had been activated after injury progressively decrease their activity until they are finished. Most of the macrophages and myofibroblasts undergo apoptosis or exit from the wound, leaving a mass that contains few cells [35].

The morphological observations allowed us to verify that repaired tissue appeared more organized than after 7 days, showing higher density of mature collagen type I. Histological assessment of the group of animals treated with hydrolyzed collagen (T2 group) showed a newly stratified epidermis, with dense and organized connective tissue presenting a great number of fibroblasts and few inflammatory cells.

Other preclinical trials using modified collagen gel treatments versus untreated wounds have shown acute inflammatory cell and fibroblast recruitment, collagen I deposition, increased endothelial cells, upregulated vascular endothelial growth factor, and improved blood flow [36,37]. In accordance with our results, clinical studies [19] report that collagen-treated wounds displayed increased neoangiogenesis, less inflammatory granulation tissue, and more organized and well-formed collagen bundles compared to the primary closure of punch biopsies with nonabsorbable sutures.

## 5. Conclusions

Taking into account all the morphometric and histological results of this research, we can conclude that the new collagen treatment, compared to the untreated control group, produces significantly faster wound closure and, at the same time, promotes a slight progression of the reparative process, showing more mature and organized—and, therefore, higher-quality—repair tissue, compared with the rest of the groups.

However, additional studies are needed to confirm the effects of this collagen-based product in terms of a compromised healing process.

## Data Availability

Data is contained within the article.
